# UCST-Activated Network Reinforcement in Hybrid Microgels for Smart Plugging

**DOI:** 10.3390/gels12010008

**Published:** 2025-12-21

**Authors:** Mingliang Du, Huifeng He, Qingchen Wang, Keming Sheng, Guancheng Jiang, Yinbo He

**Affiliations:** 1 State Key Laboratory of Petroleum Resources and Prospecting, China University of Petroleum (Beijing), Beijing 102249, China; 2 College of Petroleum Engineering, China University of Petroleum (Beijing), Beijing 102249, China; 3 Changqing General Drilling Company, Chuanqing Drilling Engineering Company of CNPC, Xi’an 710018, China; 4 College of Information Science and Engineering/College of Artiffcial Intelligence, China University of Petroleum (Beijing), Beijing 102249, China; 5 Hainan Institute of China University of Petroleum (Beijing), Sanya 572000, China

**Keywords:** stimuli-responsive, hybrid microgel, UCST, dynamic network reinforcement, high-temperature plugging, lost circulation material

## Abstract

Conventional polymer-based plugging materials often fail in deep-well environments due to passive thermal softening and network relaxation, which significantly compromise mechanical integrity and interfacial retention. To address this challenge, a novel smart Upper Critical Solution Temperature (UCST)-responsive hybrid microgel (SUPA) was synthesized for adaptive plugging in complex formations. The distinctive UCST responsiveness was conferred by incorporating N-(2-amino-2-oxoethyl)acrylamide (NAGA) and N-(2-hydroxypropyl) methacrylamide (HPMA) functional units into a robust dual-crosslinked network. Particle size analysis and oscillatory rheology in saline solution revealed the thermal activation mechanism: surpassing the critical temperature triggers the dissociation of intramolecular hydrogen bonds, driving polymer chain extension and volumetric expansion. This conformational transition induces dynamic network reinforcement, quantified by a significant ~7.5-fold increase in the storage modulus (G′). Consequently, the SUPA-enhanced fluid exhibited superior rheological performance, including a 4.4-fold increase in low-shear viscosity and rapid thixotropic recovery (ratio of 1.06). Crucially, lost circulation tests confirmed reliable and highly efficient sealing performance under harsh conditions of 150 °C and 5 MPa, even in fractured models. This study validates a design strategy centered on UCST-activated network reinforcement, offering a robust, mechanism-driven solution for severe lost circulation control in deep-well drilling.

## 1. Introduction

Drilling fluid is essential for drilling operations, performing critical functions such as maintaining formation pressure balance, effective cuttings transport, lubrication, and cooling of the drill bit and string [1,2,3]. It is also instrumental in mitigating downhole complications, including lost circulation (LC), wellbore collapse, pipe sticking, and reservoir damage [4]. Lost circulation represents one of the most persistent and costly challenges encountered during drilling. It leads to substantial drilling fluid loss, increases non-productive time (NPT), and elevates risks associated with well control issues (e.g., blowouts), wellbore instability, and stuck pipe incidents [5]. Globally, LC is estimated to cause billions of dollars in annual economic losses, serving as a primary constraint on drilling efficiency and safety [6]. Recent modeling studies have further highlighted the complexity of wellbore strengthening and the critical role of poroelastoplastic effects in fracture closure, underscoring the need for advanced materials capable of adapting to dynamic stress environments [7,8].

Plugging operations are generally categorized as static (to seal major fractures or vugs, often requiring cessation of drilling) and dynamic (Lost Circulation Material, LCM, incorporated into the drilling fluid to seal seepage loss and micro-fissures while drilling proceeds) [9]. Gel-based plugging agents have gained prominence in dynamic LC control due to their intrinsic flexibility and deformability, allowing them to adapt effectively to multi-scale and irregular loss pathways without stringent reliance on particle-fracture size matching [10,11,12]. However, the application of conventional polymer-based gels in high-temperature, high-pressure (HPHT) environments is severely restricted by their inherent thermal softening and network relaxation characteristics [13]. Instead of maintaining strength, the viscoelasticity and mechanical integrity of these materials passively decline as temperature rises [14,15]. This leads to a substantial reduction in interfacial adsorption capacity and a weakened network elastic modulus, making the sealing layer vulnerable to erosion and extrusion failure under thermal stress [16,17]. This passive performance reduction constitutes a critical bottleneck, creating an irreconcilable conflict between surface pumpability and downhole sealing strength [18,19,20,21]. Consequently, the development of novel smart gel materials is imperative [22]. Such materials must offer adaptive sealing advantages: maintaining low viscosity and pumpability at lower wellbore sections, compatibility with solids control equipment, and, crucially, precise activation in the high-temperature loss zone to realize dynamic network enhancement and self-adaptive densification for the formation of a high-strength, pressure-resistant sealing barrier.

Temperature-responsive gel materials have garnered significant attention across various fields, including biomedicine and environmental engineering [23,24,25,26,27], due to their ability to undergo rapid and reversible functional transitions at specific thermal thresholds. In the context of HPHT plugging, Upper Critical Solution Temperature (UCST) responsive hydrogels offer an ideal adaptive solution [28,29,30]. The molecular mechanism of UCST systems dictates that below the critical temperature (*T*c), the polymers typically remain in a compact, coiled conformation, thereby maintaining favorable flow properties. Upon heating above the critical point, thermal energy is utilized to disrupt intramolecular hydrogen bonds, which drives polymer chain expansion [31]. This conformational change enhances hydrophilicity and polymer-solvent interaction, leading to thermally-induced gelation, increased adsorption, or network reinforcement. This unique high-temperature activation characteristic provides a distinct advantage in demanding deep-well operations.

In this study, we present a novel design strategy for a smart UCST-responsive hybrid microgel (SUPA), synthesized via bulk polymerization followed by mechanical pulverization to yield robust micro-sized particulates. The specific UCST responsiveness was engineered through the incorporation of NAGA and HPMA functional units, while a dual-crosslinked network combining organic covalent bonds with inorganic Si–O–Si hybridization was employed to ensure structural integrity, achieving an onset degradation temperature exceeding 270 °C. Variable-temperature UV-Vis spectroscopy, particle size analysis, and oscillatory rheology confirmed that the dissociation of intramolecular hydrogen bonds above the critical temperature drives polymer chain extension and dynamic network reinforcement. Crucially, intrinsic rheological characterization in a clay-free saline environment verified that the observed ~7.5-fold increase in storage modulus (G′) is a fundamental attribute of the polymer network itself, independent of clay interactions. Furthermore, the SUPA system demonstrated superior rheological performance in high-salinity field fluids. Comprehensive lost circulation tests, including fracture simulations with shut-in periods, validated reliable and highly efficient sealing performance at 150 °C and 5 MPa. Collectively, this work proposes an advanced strategy for constructing high-performance thermosensitive hybrid microgels, offering a robust, mechanism-driven solution for mitigating severe fluid loss in complex deep-well environments.

## 2. Results and Discussion

### 2.1. Molecular Design and Structural Confirmation

#### 2.1.1. Molecular Design Strategy of SUPA

**Figure 1:**
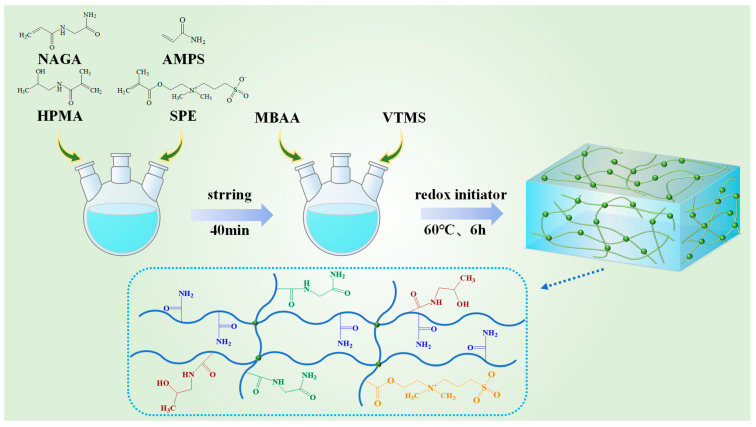
Schematic illustration of the synthesis route and rational molecular design strategy of the SUPA.

The molecular architecture of SUPA was rationally designed via free-radical random copolymerization to bridge the gap between two conflicting engineering requirements in deep-well drilling: surface screenability (necessitating low viscosity and compact conformation) and downhole sealing integrity (demanding high mechanical strength and robust interfacial retention). As illustrated in Figure 1, by integrating four functional monomers into a covalent-inorganic dual-crosslinked network, specific chemical moieties were engineered to play synergistic roles in the proposed “UCST-activated network reinforcement” mechanism. The rationale for selecting each monomeric unit is detailed as follows:

The “Smart” Thermos-Switch (NAGA): N-(2-amino-2-oxoethyl)acrylamide (NAGA) functions as the primary UCST trigger. At lower surface temperatures, the pendant amide groups engage in dense intramolecular hydrogen bonding, effectively sequestering the polymer chains into a curled, compact globular state. This ensures the material passes smoothly through shale shakers. Upon exposure to downhole thermal stimuli (~150 °C), these hydrogen bonds dissociate, triggering a conformational transition to an extended chain state (activation).

Interfacial Anchoring (HPMA): Complementing the chain unfolding, N-(2-hydroxypropyl)methacrylamide (HPMA) was introduced to facilitate interfacial adhesion. The extended NAGA chains, in synergy with HPMA, expose a high density of amide and hydroxyl groups. These polar moieties act as “molecular anchors,” creating strong hydrogen bonds and physical adsorption sites on the rock surface to prevent slippage.

Zwitterionic Salinity Resistance (SPE): Given that formation fluids and drilling muds often contain high salinity (e.g., 8 wt% KCl), which typically screens electrostatic repulsion and suppresses swelling, the “smart” activation must be salt-tolerant. The zwitterionic monomer sulfobetaine methacrylate (SPE) was incorporated to leverage the “anti-polyelectrolyte effect.” This ensures that network reinforcement and chain extension are not compromised by high ionic strength but are preserved within the saline drilling fluid environment.

Robust Hybrid Skeleton (VTMS & MBAA): To guarantee structural integrity under high shear and pressure differentials (5 MPa) at 150 °C, a covalent-inorganic dual-crosslinking strategy was implemented. While N,N’-Methylenebisacrylamide (MBAA) provides the fundamental elastic network, vinyltrimethoxysilane (VTMS) introduces rigid Si–O–Si inorganic nodes. This hybrid skeleton imparts essential mechanical rigidity to the expanded gel, ensuring that post-activation, the material possesses sufficient strength to effectively seal pore throats and fractures.

In summary, the precise molar topology (AM:SPE:NAGA:HPMA ≈ 9.6:1:2:1.7) endows the material with a specific smart functional lifecycle: “Low-viscosity transport → Thermally-triggered activation → High-strength anchoring and sealing.”

#### 2.1.2. FTIR Analysis

**Figure 2:**
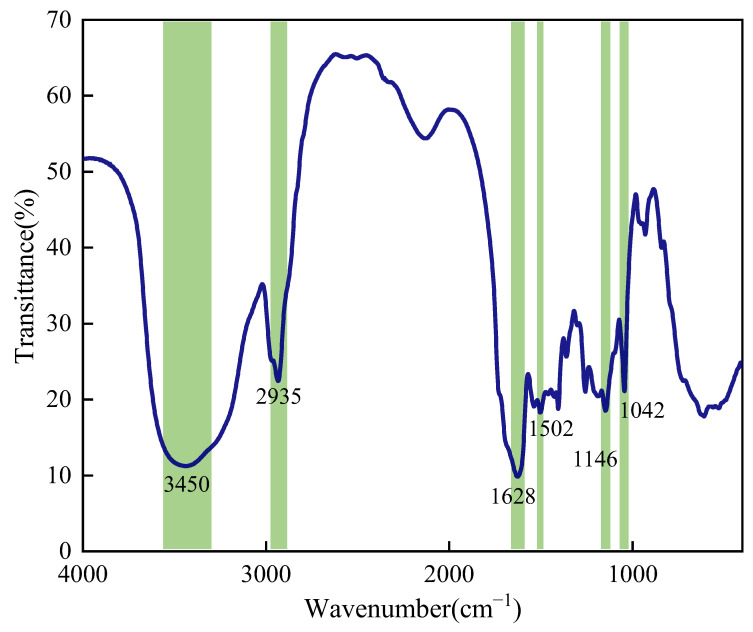
Fourier Transform Infrared (FTIR) spectrum of the synthesized SUPA copolymer, confirming the incorporation of functional groups.

The chemical structure of the synthesized SUPA copolymer was corroborated by FTIR spectroscopy (Figure 2). The spectrum exhibits characteristic absorption bands corresponding to all constituent monomers, confirming successful copolymerization. A broad, intense band centered at ~3444 cm^−1^ is ascribed to the overlapping O–H and N–H stretching vibrations from the amide groups (AM, NAGA, HPMA) and hydroxyl groups (HPMA), which suggests the presence of robust hydrogen bonding interactions within the polymer matrix. The aliphatic C–H stretching vibrations appear at 2933–2935 cm^−1^, confirming the construction of the polymer backbone.

Regarding the functional moieties, the prominent absorption band at ~1626 cm^−1^ corresponds to the Amide I (C=O stretching) vibration, while the band at 1536 cm^−1^ is assigned to the Amide II (N–H bending) vibration, verifying the presence of acrylamide-based units. In the fingerprint region, the characteristic symmetric and asymmetric S=O stretching vibrations of the sulfonate group in SPE are clearly identified at 1042 cm^−1^ and 1146 cm^−1^, respectively. Most notably, the formation of the covalent-inorganic hybrid network is evidenced by the appearance of weak bands near 800–850 cm^−1^ and 460–480 cm^−1^. These are assigned to the symmetric stretching and bending vibrations of Si–O–Si bonds derived from the condensation of VTMS. While these signals are relatively subtle, their intensity is commensurate with the optimized low dosage of VTMS (~0.5 wt%), which was deliberately controlled to provide sufficient crosslinking density without inducing excessive brittleness. Furthermore, the absence of characteristic vinyl C=C stretching peaks (typically near 1640 cm^−1^) confirms a high degree of polymerization and the effective removal of unreacted monomers during purification.

#### 2.1.3. Thermal Stability (TGA/DSC)

**Figure 3:**
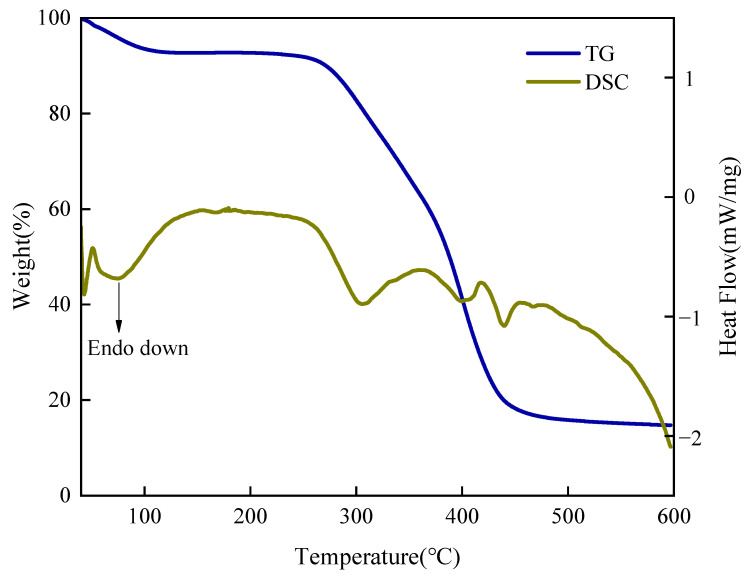
Thermogravimetric Analysis (TGA) and Differential Scanning Calorimetry (DSC) curves of SUPA, demonstrating its thermal stability.

The thermal stability of SUPA was evaluated via TGA and DSC analyses (Figure 3), revealing a three-stage thermal degradation profile. Initially (Stage I, 40–120 °C), a mass loss of ~7.1% accompanied by a broad endothermic DSC peak is observed. This is attributed to the evaporation of physically adsorbed and bound water, reflecting the strong hydrophilicity of the amide and zwitterionic groups. Subsequently (Stage II, 120–270 °C), the material exhibits a stable plateau with minimal mass loss (<0.2%), indicating excellent structural stability within the target operational temperature range of drilling fluids. Finally (Stage III, >270 °C), significant chemical degradation commences only above the onset temperature (T_onset_ ≈ 270 °C). The primary weight loss (270–450 °C) corresponds to the thermal decomposition of pendant groups followed by main chain scission. Notably, the dry-basis char yield at 600 °C is approximately 15.9%. This relatively high residue is attributed to the synergistic effect of the organic-inorganic hybrid structure: the rigid Si–O–Si network formed by VTMS acts as a thermal barrier, restricting chain mobility and promoting carbonization. In conclusion, SUPA exhibits remarkable chemical stability below 270 °C, providing reliable assurance for applications in harsh high-temperature environments.

### 2.2. Micro-Morphology and Elemental Distribution

**Figure 4:**
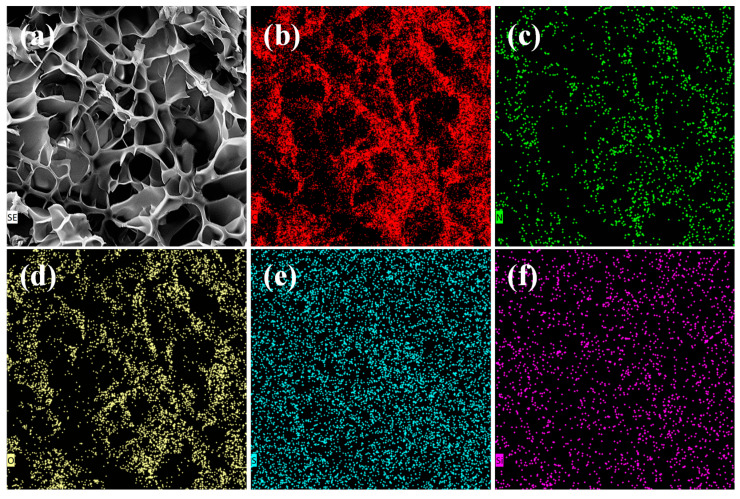
Cryo-Scanning Electron Microscopy (Cryo-SEM) morphology and Energy-Dispersive X-Ray Spectroscopy (EDS) elemental mapping of SUPA. (**a**) Microstructure of the hydrogel network (Scale bar: 20 μm); (**b**–**f**) Elemental distribution maps corresponding to (**b**) Carbon C, (**c**) Nitrogen (N), (**d**) Oxygen (O), (**e**) Sulfur (S), and (**f**) Silicon (Si).

Cryo-SEM was employed to elucidate the internal microstructure of the swollen SUPA hydrogel, offering insights into its liquid-holding capacity and network topology. As depicted in Figure 4a, SUPA exhibits a well-developed, continuous three-dimensional porous network characterized by highly interconnected channels. This open-cellular morphology is instrumental in the material’s “smart” mechanism, as it facilitates the rapid diffusion of water molecules, thereby enabling the substantial volumetric expansion required for effective plugging upon activation.

**Figure 5:**
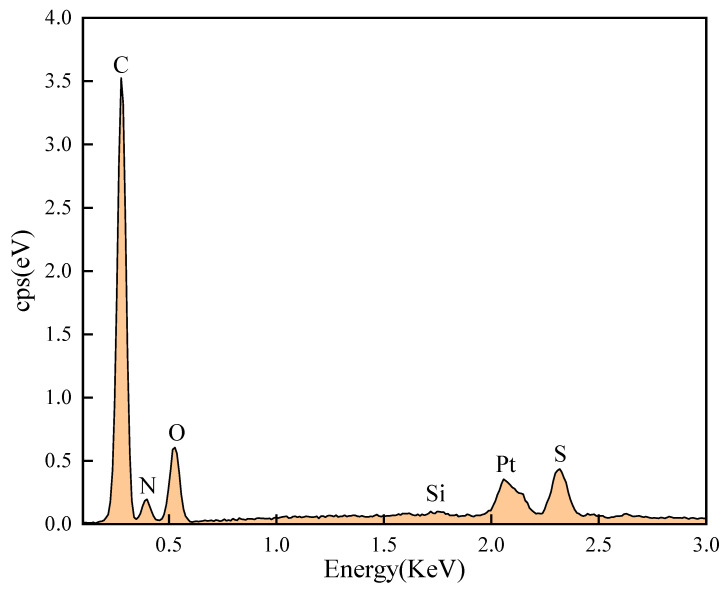
Quantitative Elemental Composition Analysis of SUPA determined by EDS.

Complementing the morphological analysis, EDS was performed to confirm the chemical composition and homogeneity (Figure 5). The spectrum reveals prominent peaks for C, O, and N, which constitute the organic backbone. Notably, distinct signals for S (attributed to the sulfonate group in SPE) and Si (derived from the silane crosslinker VTMS) are clearly identified. While the Si signal intensity is modest—commensurate with the strategic low dosage (~0.5 wt%) intended to balance crosslinking density with ductility—its detection unequivocally confirms the successful incorporation of inorganic nodes.

Furthermore, elemental mapping images (Figure 4b–f) demonstrate that both Sulfur (S) and Silicon (Si) are uniformly distributed throughout the Carbon matrix without localized aggregation. This homogeneous integration suggests that the monomers were successfully copolymerized into a random network rather than forming phase-separated blocky structures. Such structural uniformity is pivotal for ensuring isotropic salt tolerance and mechanical stress distribution across the gel matrix.

### 2.3. Thermo-Responsive Mechanism and Activation Behavior

#### 2.3.1. Molecular Conformation Transition (Variable-Temp UV-Vis)

**Figure 6:**
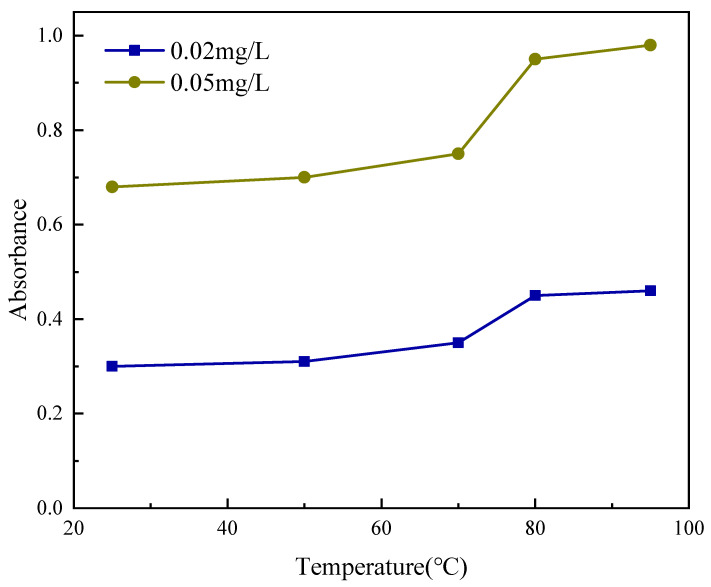
Variable-temperature UV-Vis spectra of SUPA dispersion (absorbance at 252 nm) revealing the molecular conformation transition.

To elucidate the molecular driver of thermal activation, variable-temperature UV-Vis spectroscopy was employed to monitor the absorbance evolution of the amide carbonyl (C=O) n → π* transition at 252 nm. An ultra-dilute concentration was strictly utilized to minimize potential interference from light scattering, ensuring the signal primarily reflects the local conformational state of the polymer chains (Figure 6). Below 70 °C, the absorbance remains relatively low and stable, indicating that the NAGA-based segments adopt a tightly coiled, “globule-like” conformation. In this collapsed state, stabilized by dense intramolecular hydrogen bonds, the amide chromophores are effectively sequestered within the hydrophobic core, resulting in steric shielding from the aqueous solvent.

As the temperature traverses the 70–80 °C range (Activation Zone), a pronounced sigmoidal increase in absorbance is observed. This transition signifies the thermal disruption of intramolecular hydrogen bonds, triggering a coil-to-globule transition. Consequently, the polymer chains unfold, exposing the previously shielded hydrophilic C=O groups to the surrounding water. This exposure alters the local dielectric environment of the chromophores, thereby enhancing their molar extinction coefficient—a phenomenon known as the hyperchromic effect [32,33].

It is noteworthy that simple particle swelling typically reduces turbidity (and thus apparent absorbance) due to the diminished refractive index contrast between the hydrated gel and the solvent. Conversely, the observed increase in absorbance confirms that the spectral evolution is dominated by the molecular hyperchromic effect rather than scattering artifacts [34]. Furthermore, the onset temperature of this transition (T ≈ 74 °C) aligns precisely with the critical thresholds observed in both particle size expansion (Section 2.3.2) and rheological stiffening (Section 2.3.3). This convergence of spectroscopic, dimensional, and rheological data provides robust evidence that the macroscopic property changes are fundamentally driven by the hydrogen-bond-mediated molecular conformational transition.

#### 2.3.2. Particle Size Evolution and Swelling Kinetics

**Figure 7:**
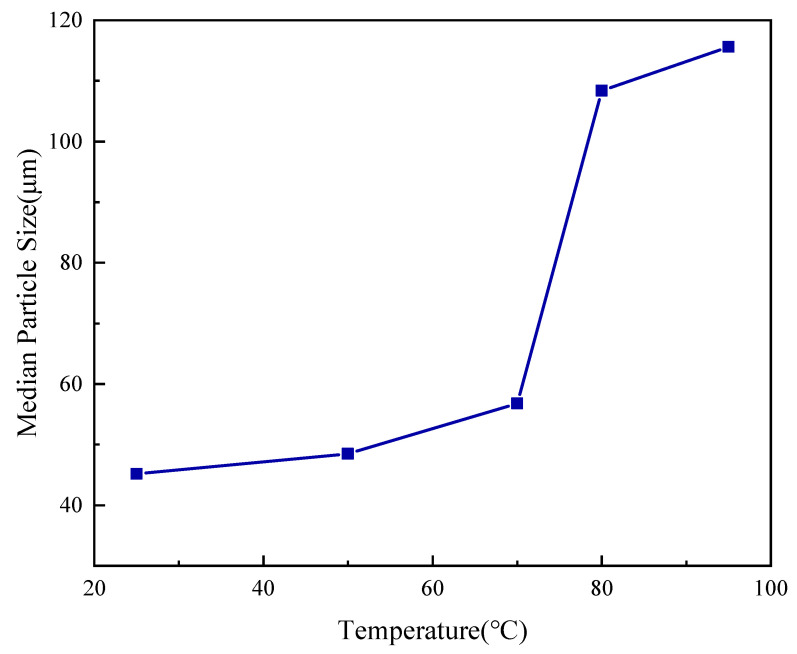
Median Particle Size of SUPA microgels as a function of temperature (25–95 °C).

The temperature-dependent swelling behavior, quantified by median particle size (D_50_), corroborates the UV-Vis findings (Figure 7). During the Surface-Stable Phase (25–70 °C), the particle size increases marginally (45.2 → 56.8 µm). This “dormant” state is engineered for operational efficiency, ensuring compact microgels pass through surface shale shakers (>75 µm mesh) without premature removal. However, a Thermally-Triggered Expansion occurs between 70 °C and 95 °C. A sharp inflection aligns with the UCST activation zone, where D_50_ surges to 108.4 µm. At 95 °C, the particle size stabilizes at ~115.6 µm. Quantitatively, SUPA microgels achieve a ~2.56-fold increase in diameter (~16.7-fold in volume) when heated to reservoir temperatures. This “in-situ expansion” allows the material to enter fractures as small particles and then expand to form a tight seal once activated by the geothermal gradient.

#### 2.3.3. Intrinsic Viscoelastic Phase Transition in Saline Solution

**Figure 8:**
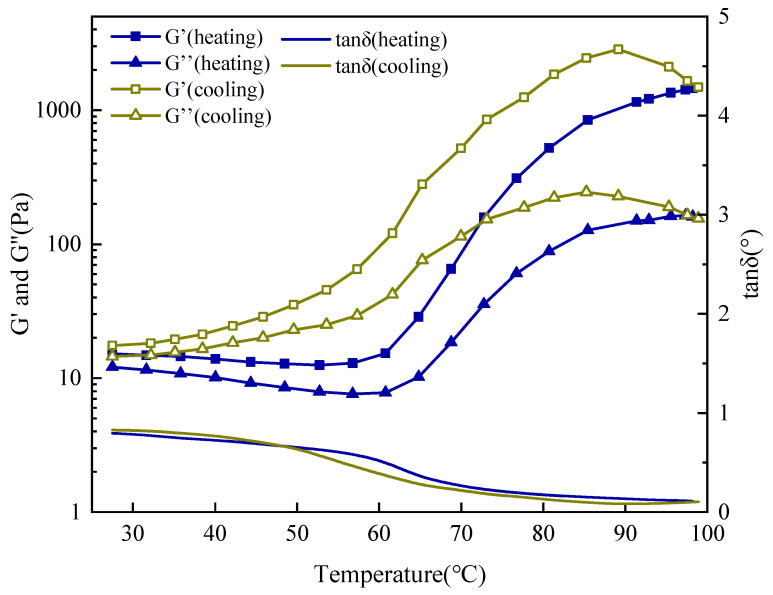
Oscillatory temperature sweep rheology of 1 wt% SUPA in 5 wt% KCl solution, demonstrating the intrinsic UCST viscoelastic transition without clay interference.

To verify the intrinsic thermo-responsiveness of the SUPA polymer—independent of the complex drilling fluid matrix—oscillatory temperature sweep tests were performed in a particle-free model saline solution (5 wt% KCl). As illustrated in Figure 8, the system exhibits a characteristic thermo-thickening behavior, confirming that the UCST attributes are inherent to the polymer network architecture.

In the low-temperature regime (25–55 °C), the solution demonstrates sol-like characteristics, defined by a low storage modulus (G′ ≈ 13–15 Pa) and a high loss factor (tan δ ≈ 0.7–0.8). A dramatic rheological transition is triggered at ~60 °C, where G′ surges by two orders of magnitude, culminating in a plateau of ~1480 Pa at 99 °C. The concomitant drop in tan δ to 0.10 signifies the evolution into a robust, solid-like elastic network. These results conclusively prove that the structural strengthening is driven by the polymer’s conformational unfolding and salt-tolerant zwitterionic associations, rather than extrinsic interactions with clay particles.

Significantly, the cooling profile demonstrates excellent reversibility with minimal hysteresis. Upon cooling to 29 °C, G′ recovers to 17.5 Pa, virtually returning to its initial state. This reversible “Heat-Thickening/Cool-Thinning” attribute confers a vital operational safety margin: it ensures that while the gel forms a rigid plug at elevated formation temperatures, the fluid reverts to a flowable, low-viscosity state within the cooler wellbore or during circulation. This rheological adaptability is crucial for preventing accidental gelation and mitigating blockage risks in drill strings and surface equipment.

### 2.4. Rheological Enhancement in Drilling Fluid Systems

#### 2.4.1. UCST-Induced Network Reinforcement in Drilling Fluid

**Figure 9:**
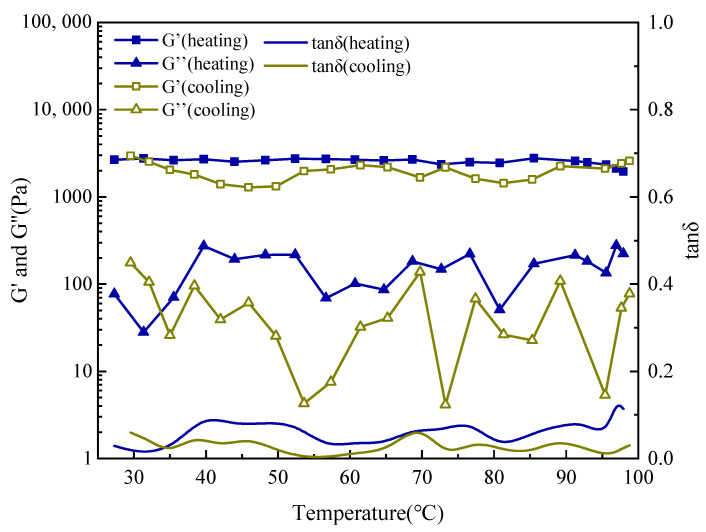
Oscillatory temperature sweep rheology of the pristine field drilling fluid.

**Figure 10:**
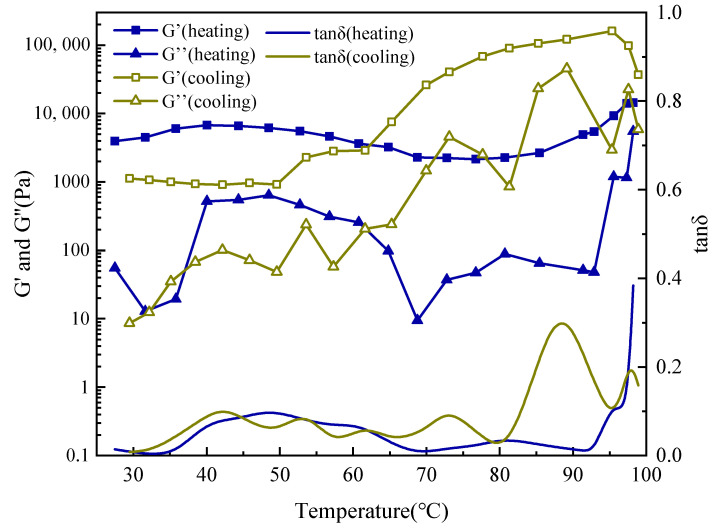
Oscillatory temperature sweep rheology of the field drilling fluid containing 1 wt% SUPA, demonstrating UCST-induced network reinforcement.

To assess the performance of SUPA under simulated downhole conditions, dynamic rheological measurements were performed on a field-derived water-based drilling fluid (formulated with 8 wt% KCl) in the presence and absence of 1 wt% SUPA (Figure 9 and Figure 10). The unadulterated base fluid (Control, Figure 9) exhibits behavior typical of a thermally stable but non-responsive suspension, characterized by monotonic thermal thinning. Specifically, the storage modulus (G′) gradually declines from ~2673 Pa at 27.3 °C to ~1954 Pa at 97.9 °C, confirming the lack of intrinsic thermo-responsive mechanisms in the base formulation.

In stark contrast, the incorporation of 1 wt% SUPA endows the system with a pronounced UCST-type thermo-thickening capability (Figure 10). Following an initial thermal softening regime (27–72 °C)—attributable to enhanced polymer chain mobility—the system undergoes a distinct rheological transition upon crossing the critical temperature threshold (Tc ≈ 74–76 °C). Here, G′ exhibits a sharp upturn, escalating from ~4903 Pa to a peak of 37,030 Pa at 98.9 °C. This ~7.5-fold surge within a narrow temperature window serves as the definitive rheological signature of UCST gelation, driven by the thermal dissociation of intramolecular hydrogen bonds and the subsequent establishment of a rigid, interactive polymer-particle network.

Furthermore, the cooling trajectory reveals significant thermal hysteresis, with G′ attaining a transient maximum of ~160,700 Pa at 95.3 °C—representing a ~60-fold enhancement relative to the base fluid. This hysteresis is characteristic of hydrogen-bond-dominated networks interacting with clay platelets, where cooling initially promotes additional crosslinking points before chain relaxation occurs. Crucially, despite this transient strengthening, the system does not undergo irreversible curing. As the temperature returns to ambient conditions, G′ recedes to a lower modulus state (~900 Pa). While this residual value is higher than the initial state due to structural reorganization, it remains well within the range of pumpable fluid consistency. This confirms that the “smart” network retains its fundamental reversibility even within a complex solid-laden matrix, ensuring the fluid can form a competent seal at elevated formation temperatures while reverting to a manageable, low-viscosity state at surface conditions.

#### 2.4.2. Shear Thinning Analysis

**Figure 11:**
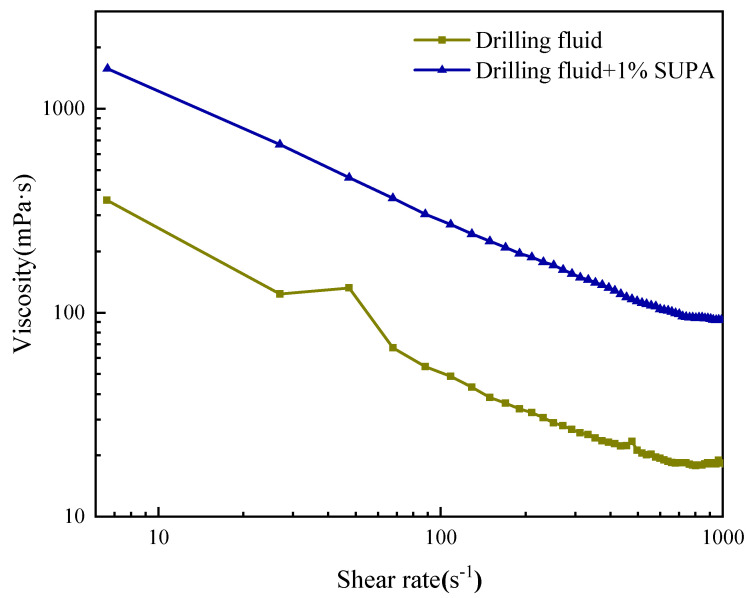
Steady-state shear rheology: Viscosity and shear stress curves of the drilling fluids as a function of shear rate.

Steady-state shear tests (Figure 11) provide critical insights into how SUPA modifies the flow behavior of the drilling fluid. The addition of 1 wt% SUPA significantly elevates the entire viscosity profile while strictly preserving the desirable shear-thinning characteristic, as evidenced by the stability of the flow behavior index (n ≈ 0.44) derived from Power Law fitting. Quantitatively, the system exhibits a dual-response mechanism: at high shear rates (1021 s^−1^), the viscosity increases by 5.1-fold (18.1 → 92.5 mPa·s), whereas at low shear rates (6.6 s^−1^), it surges by 4.4-fold (356.5 → 1571 mPa·s). Concurrently, the consistency coefficient (K) rises significantly from 1.08 to 4.51 Pa·s^n^.

This rheological architecture implies that SUPA strengthens the internal network via multi-point anchoring—facilitated by hydrogen bonding and electrostatic interactions from zwitterionic groups—without creating a rigid, non-flowable structure. This result is pivotal for engineering safety and efficiency: it confirms that despite the thermally induced viscosity increase, the fluid retains excellent pumpability within the drill pipe and bit nozzles (high shear zones). Simultaneously, the elevated low-shear viscosity ensures high carrying capacity in the annulus, effectively transporting cuttings even at lower annular velocities.

#### 2.4.3. Thixotropic Response

**Figure 12:**
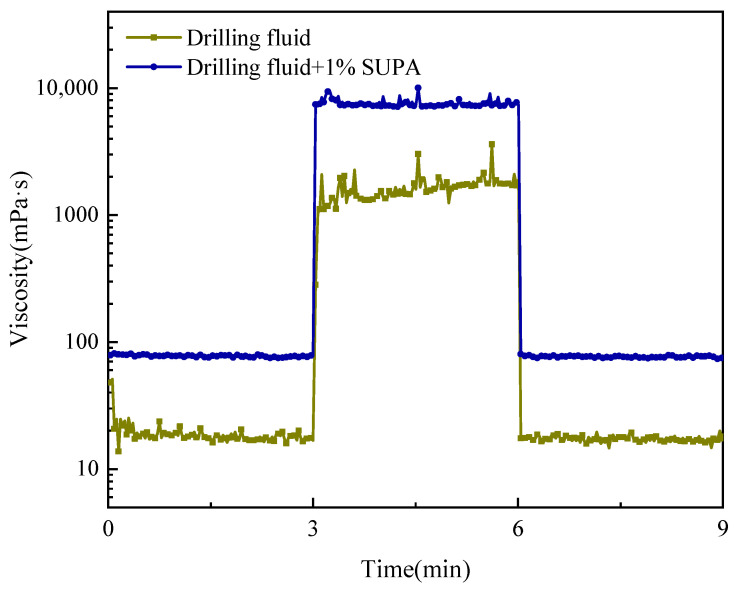
Three-interval thixotropy test (3ITT) results showing the structural breakdown and recovery kinetics of the drilling fluids.

The three-interval thixotropy test (3ITT, Figure 12) highlights the superior structural recovery kinetics of the SUPA system, which is paramount for managing downhole pressure profiles [35,36]. During the initial high-shear phase, although the SUPA system maintains a higher baseline viscosity (76–80 mPa·s) compared to the control (~18 mPa·s), it demonstrates rapid structural breakdown, consistent with the shear-thinning results. The critical performance differentiator emerges upon the cessation of shear. When switched to a low shear rate (1 s^−1^), the SUPA-enhanced fluid rebuilds to a viscosity of 7745 mPa·s within a mere 10 s. This corresponds to a recovery ratio of 1.06, indicating a slight structural overshoot where the reformed network is momentarily stronger than the original state. In sharp contrast, the control fluid recovers sluggishly and only to a ratio of 0.58.

This rapid, second-scale recovery capability provides a definitive answer to concerns regarding thermal hysteresis. While temperature sweeps indicated a hysteresis loop (retaining high viscosity upon cooling), the thixotropic data confirms that this “thickened” state is not permanent but is dynamically responsive to shear. This ensures a vital safety margin: when circulation stops (e.g., for pipe connections or tripping), the fluid instantly forms a robust gel structure to suspend cuttings and seal micro-fractures, preventing sedimentation and fluid loss. Conversely, as soon as circulation resumes, the structure breaks down easily, mitigating pressure surges and ensuring restart ability.

### 2.5. High-Temperature Lost Circulation Control Performance

#### 2.5.1. Pore Sealing Capabilities (Dynamic Sand Bed Tests)

**Figure 13:**
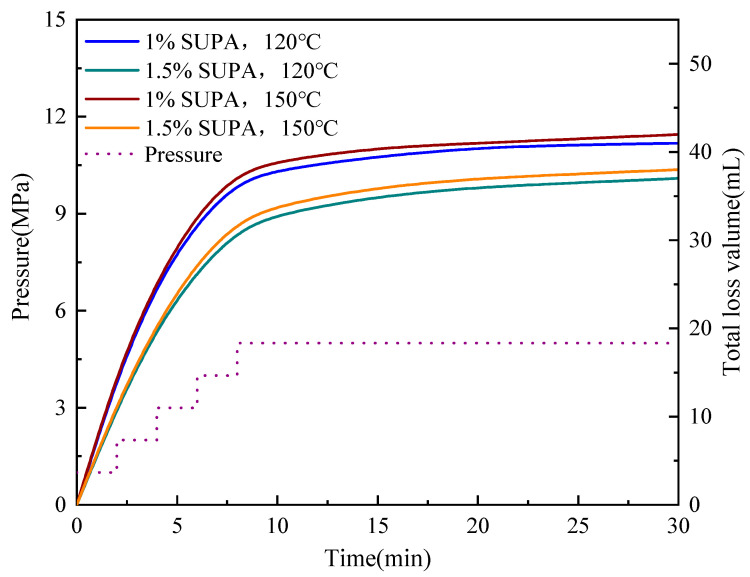
Dynamic lost circulation performance of SUPA systems in a fine sand bed (40–60 mesh).

**Figure 14:**
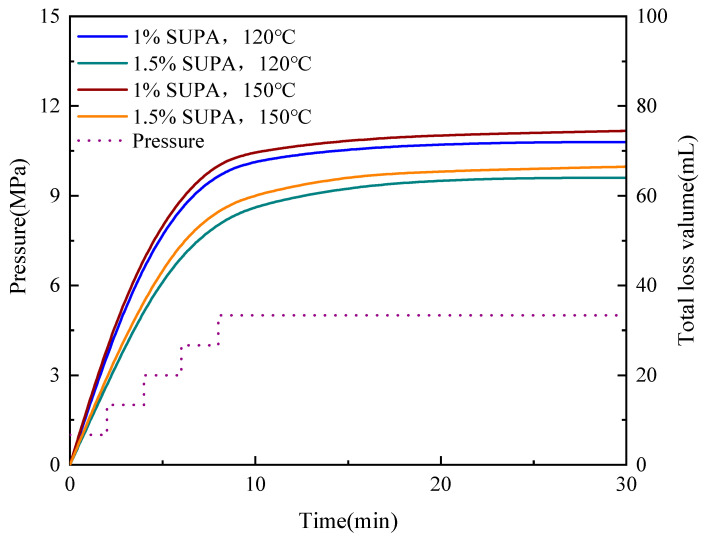
Dynamic lost circulation performance of SUPA systems in a coarse sand bed (20–40 mesh).

**Figure 15:**
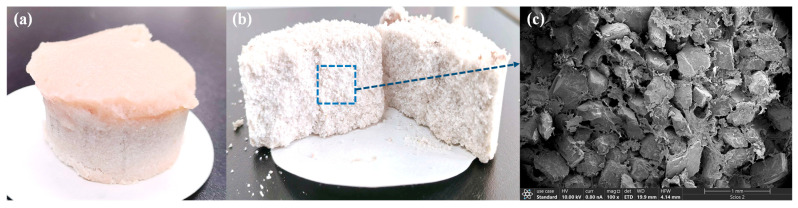
Post-test characterization of the consolidated sand bed. (**a**) Macroscopic morphology of the consolidated sand bed; (**b**) Cross-sectional view showing the structural integrity of the plugging layer; (**c**) SEM micrograph revealing the dense microstructural bonding between SUPA hydrogels and sand grains.

The plugging efficacy of the SUPA system under simulated High-Pressure High-Temperature (HPHT) conditions was evaluated using dynamic sand bed filtration tests at 5 MPa and temperatures of 120 °C and 150 °C (Figure 13 and Figure 14). The SUPA gel exhibited exceptional sealing capabilities, particularly at the optimized concentration of 1.5 wt%. In the fine sand bed (40–60 mesh), the 30-min cumulative fluid loss (V_30_) remained remarkably low and stable (37.0–38.0 mL). Even in the medium sand bed (20–40 mesh) with higher permeability, the system maintained robust pressure-bearing capacity, controlling V_30_ values between 64.0 and 66.5 mL. A critical finding here is the temperature insensitivity of the sealing performance; the fluid loss at 150 °C was virtually identical to that at 120 °C. This counterintuitive stability is directly attributed to the UCST activation mechanism: while elevated temperatures typically induce thermal thinning in conventional fluids, the thermal energy (>T_c_ ≈ 76 °C) in the SUPA system disrupts intramolecular hydrogen bonds. This exposes active amide and hydroxyl groups, enhancing chemical adsorption and polymer bridging onto sand grain surfaces, which effectively counteracts the viscosity reduction. This mechanism is visually corroborated by post-test SEM analysis (Figure 15), which reveals that the SUPA gel forms a continuous, cohesive phase that firmly bonds loose sand grains into a dense, impermeable barrier.

**Figure 16:**
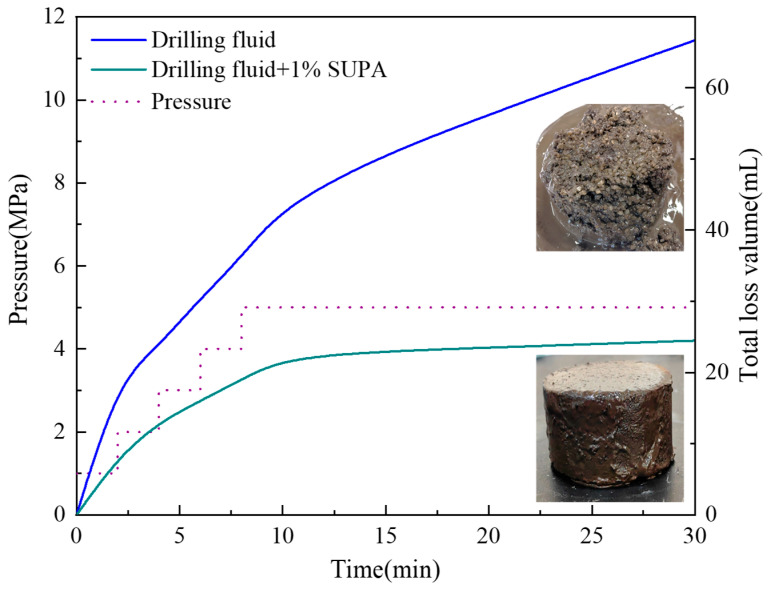
Dynamic lost circulation performance of SUPA systems in a coarse gravel bed (10–20 mesh).

To further challenge the bridging capability, tests were conducted in a highly permeable coarse gravel bed (10–20 mesh) at 150 °C (Figure 16). The pristine drilling fluid failed to establish an effective seal, exhibiting a linear increase in cumulative loss with time (V_30_ = 66.7 mL), indicating uncontrolled filtration. In sharp contrast, the addition of 1 wt% SUPA dramatically improved performance through a two-stage mechanism. Initially, the swollen microgels rapidly bridged large pore throats, curbing fluid loss to 22 mL during the first 10 min (vs. 43.4 mL for the control). Most notably, the system achieved a “leak-off arrest” phenomenon after 15 min, where the cumulative loss curve reached a distinct plateau with only a marginal increment of 1.5 mL between 15 and 30 min. This confirms that the high-temperature activated microgels do not merely increase viscosity but interlock within pore throats and adhere to gravel surfaces, forming a structurally rigid, pressure-bearing plug that effectively shuts off flow channels even in unconsolidated, high-permeability formations.

#### 2.5.2. Fracture Sealing Efficiency (Slotted Plate Tests)

**Figure 17:**
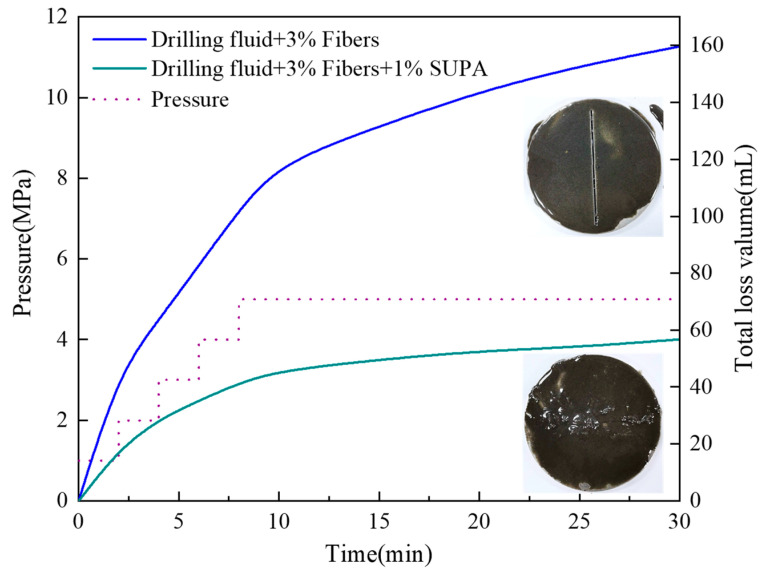
Sealing efficiency of the SUPA system evaluated using a 1 mm slotted plate.

Sealing macroscopic fractures presents a severe hydrodynamic challenge compared to pore plugging due to the negligible tortuosity of flow channels. To evaluate the plugging performance of SUPA under these conditions, dynamic filtration tests were performed using a steel plate with a 1 mm slot to simulate an open fracture, employing commercial mineral fibers (average length ~200 µm, 3 wt%) as the bridging agent. The protocol included a thermal aging phase (150 °C, 1 MPa for 1 h) followed by a stepped pressure ramp (1–5 MPa), as shown in Figure 17. The control fluid containing only fibers exhibited negligible sealing capacity, with cumulative losses reaching 159.6 mL after 30 min. This failure is mechanically attributed to a severe geometric mismatch: without a stabilization mechanism, the discrete fibers were insufficient to bridge the 1000 µm aperture and were continuously eluted through the slot under pressure.

In distinct contrast, the incorporation of 1 wt% SUPA reduced the total fluid loss to 56.8 mL, representing a 64.4% reduction. This performance enhancement is governed by an adsorption-assisted gelation mechanism. Upon heating above the UCST, SUPA chains unfold to expose abundant active sites that facilitate multi-site adsorption onto both mineral fibers and fracture surfaces, significantly enhancing the retention capability of the solid phase. Simultaneously, the polymer undergoes in-situ gelation, forming a cohesive structure that locks the bridged fibers in place. Consequently, the SUPA-fiber composite demonstrated robust pressure resilience during the subsequent ramp to 5 MPa with minimal incremental loss, confirming the transformation of loose fiber packs into pressure-bearing sealing layers capable of sealing fractures significantly larger than individual particle sizes.

### 2.6. Mechanism for Smart Plugging

**Figure 18:**
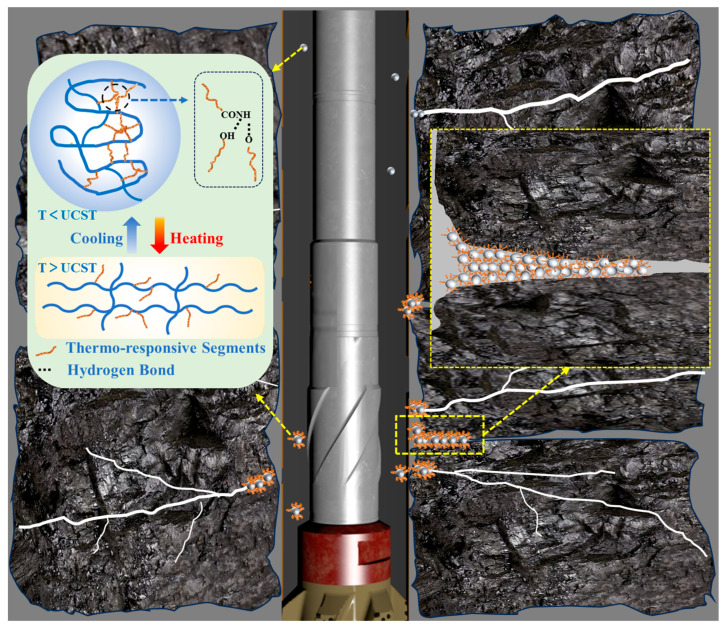
Schematic illustration of the proposed “Smart Plugging” mechanism driven by UCST-activated network reinforcement.

Synthesizing evidence from molecular spectroscopy, rheological profiling, and macroscopic fluid loss experiments, we propose a multi-scale mechanism governing the smart plugging behavior of SUPA (Figure 18). The core driver of this system is a thermally induced conformational transition from a contracted globule state to an extended coil state. Below the UCST, thermo-responsive segments are stabilized in a collapsed conformation by dense intramolecular hydrogen bonds. Consequently, the polymer presents a coiled structure with limited exposed adsorption groups and a small hydrodynamic size. This results in a weak thickening effect, ensuring optimal pumpability and injectability during surface handling and transport through the wellbore.

Upon heating above the UCST, thermal energy disrupts these intramolecular interactions, triggering chain unfolding. As corroborated by UV-Vis analysis, this conformational switch exposes abundant carbonyl (C=O) and hydroxyl (-OH) active sites. Crucially, this molecular activation translates into macroscopic seal integrity through two synergistic pathways: enhanced adhesion and structural cohesion. On one hand, the exposed polar groups function as high-density “molecular anchors,” establishing robust hydrogen bonding and electrostatic interactions with negatively charged formation minerals (e.g., silica, clays). This significantly enhances the retention capability of the plugging materials.

Simultaneously, the hydrophilic domains facilitate strong inter-particle adsorption, binding adjacent gel particles and solid bridging materials into a dense, impermeable sealing layer. Unlike conventional plugging agents that undergo thermal softening, SUPA exhibits distinct “heat-thickening” behavior. By forming a robust composite structure that effectively resists deformation and extrusion, this synergistic “Adhesion-Cohesion” efficacy validates UCST-activated network reinforcement as a core strategy for achieving reliable smart plugging in extreme high-temperature environments [37].

## 3. Conclusions

In this study, a novel covalent-inorganic dual-crosslinked microgel (SUPA) exhibiting Upper Critical Solution Temperature (UCST) responsiveness was developed to resolve the conflict between surface pumpability and downhole sealing integrity in deep-well drilling. The main conclusions are summarized as follows:

(1) A novel covalent-inorganic dual-crosslinked network was successfully constructed by integrating thermo-responsive NAGA and adhesive HPMA monomers into a hybrid skeleton reinforced by VTMS-derived silane nodes. This specific molecular topology endows the microgel with exceptional thermal stability, exhibiting an onset degradation temperature of ≥270 °C, which ensures operational reliability under high-temperature deep-well conditions.
(2) The microgel exhibits a distinct UCST phase transition at 74–76 °C, where thermal energy disrupts intramolecular hydrogen bonds. This mechanism triggers a conformational change from a coiled state to an extended state, resulting in a significant volumetric expansion where the median particle size increases from ~56.8 μm to ~115.6 μm. This microscopic evolution translates into macroscopic network reinforcement, evidenced by a ~7.5-fold increase in the storage modulus (G′) of the drilling fluid system upon heating. Intrinsic rheology in saline solution confirmed that this strengthening is a property of the polymer itself.
(3) Incorporating 1 wt% SUPA optimizes the rheological profile of high-salinity water-based drilling fluids by increasing low-shear viscosity 4.4-fold for improved cutting transport while preserving shear-thinning behavior. Furthermore, the system demonstrates rapid thixotropic recovery (rebuilding structure within 10 s), which mitigates thermal hysteresis risks and ensures suspension stability during circulation stops.
(4) Under HPHT conditions (150 °C, 5 MPa), SUPA demonstrated superior sealing capabilities across diverse formation types. In permeable sand and gravel beds, the activated microgels counteracted thermal thinning through enhanced adsorption and pore-throat interlocking, successfully cutting off leakage channels to stabilize fluid loss even in coarse gravel. Furthermore, in simulated fractured formations (1 mm slot) subjected to shut-in periods, SUPA exhibited a “Soft-Rigid Synergistic Coupling” effect with rigid mineral fibers. The swollen hydrogel cooperatively bridged wide apertures with the fibers, reducing fluid loss by 64.4% compared to fibers alone.
(5) Ultimately, the comprehensive performance of SUPA is intrinsically governed by its reversible molecular conformation. Polymer chains remain coiled and hydrogen-bond-stabilized at surface temperatures for smooth pumpability, but unfold and extend at downhole temperatures to anchor onto formation surfaces. This “Low-Viscosity Transport → High-Temperature Activation” lifecycle validates SUPA as a promising intelligent material for complex deep drilling operations.

## 4. Materials and Methods

### 4.1. Reagents and Instruments

The chemical reagents utilized in this study included Acrylamide (AM), Sulfobetaine Methacrylate (SPE), N-(2-Amino-2-oxoethyl)acrylamide (NAGA), and N-(2-Hydroxypropyl)methacrylamide (HPMA). The crosslinking agents were N,N’-Methylenebisacrylamide (MBAA) and Vinyltrimethoxysilane (VTMS). The redox initiator system was composed of Ammonium Persulfate (APS) and Sodium Hydrogen Sulfite (SHS). Additionally, deionized water was used as the solvent, and dilute aqueous ammonia and dilute hydrochloric acid were employed as pH-adjusting agents. All reagents were purchased from Aladdin Reagent Co., Ltd. (Shanghai, China) with analytical grade purity and used without further purification. Sodium bentonite and field drilling fluid additives were obtained from China Petroleum Chuanqing Drilling Engineering Company Limited (Chengdu, China).

The primary equipment included a high-speed mechanical stirrer, a constant-temperature water bath, an inert gas supply system, a constant-temperature oven, and a mechanical pulverizer. Material characterization was performed using a Magna-IR 560 Fourier Transform Infrared (FTIR) spectrometer (Nicolet, Madison, WI, USA), a NETZSCH STA 499 F5 simultaneous thermal analyzer (NETZSCH, Selb, Germany), and a Cryo-Scanning Electron Microscope (Cryo-SEM) equipped with an Energy-Dispersive X-Ray Spectroscopy (EDS) detector (FEI, Hillsboro, OR, USA). Particle size distribution was analyzed using a Mastersizer 3000 Laser Diffraction Particle Size Analyzer (Malvern Panalytical, Malvern, UK). Rheological measurements were conducted on a Haake MARS III controlled-stress rheometer (Thermo Fisher Scientific, Karlsruhe, Germany). Performance evaluation utilized a Model FA-1 high-temperature, high-pressure (HPHT) lost circulation testing apparatus (Qingdao Tongchun Oil Equipment Co., Ltd., Qingdao, China).

### 4.2. Synthesis of SUPA

SUPA was prepared via free-radical aqueous phase copolymerization, designed to construct a robust covalent-inorganic dual-crosslinked network. The polymer backbone was established by copolymerizing the primary monomer, acrylamide (AM), with functional monomers sulfobetaine methacrylate (SPE), N-(2-amino-2-oxoethyl)acrylamide (NAGA), and N-(2-hydroxypropyl) methacrylamide (HPMA). To achieve the desired “smart” responsiveness and mechanical integrity, the molar feed ratio was precisely controlled at AM:SPE:NAGA:HPMA ≈ 9.6:1:2:1.7 (relative to SPE).

Crucially, a dual-crosslinking strategy was employed to reinforce the network structure. This involved the synergistic use of the organic crosslinker N,N′-methylenebisacrylamide (MBAA) and the silane-based hybrid crosslinker vinyltrimethoxysilane (VTMS) at optimized concentrations. The polymerization was carried out in an aqueous medium at a moderate temperature under an inert atmosphere, initiated by a redox system. This specific protocol ensured efficient polymer chain propagation while simultaneously facilitating the in-situ hydrolysis and condensation of silane groups. The formation of stable Si–O–Si inorganic hybrid nodes within the covalent matrix effectively enhanced the overall structural stability and thermal resistance of the hydrogel. Post-synthesis, the bulk hydrogel underwent rigorous purification by immersion in deionized water to eliminate residual monomers and oligomers, followed by drying, mechanical pulverization, and sieving through a 200-mesh screen (<75 µm) to yield the final particulate product for characterization and application assessment.

### 4.3. Characterization

#### 4.3.1. Fourier Transform Infrared Spectroscopy

FTIR spectroscopy was performed using a Nicolet Magna-IR 560 Fourier Transform Infrared (FTIR) spectrometer (Nicolet, Madison, WI, USA) to confirm the functional groups and chemical structure of the synthesized SUPA samples. Dried polymer samples were prepared using the KBr pellet method. The spectral scanning range was set from 4000 to 400 cm^−1^ with a resolution of 4 cm^−1^.

#### 4.3.2. Thermogravimetric Analysis

Thermogravimetric Analysis (TGA) was conducted using a NETZSCH STA 499 F5 simultaneous thermal analyzer (NETZSCH, Selb, Germany). Samples were heated from room temperature to 600 °C at a constant heating rate of 15 °C/min under a continuous nitrogen atmosphere. The thermal stability, degradation characteristics, and decomposition mechanism of the SUPA material were comprehensively assessed by analyzing the TGA curve and the synchronously obtained Differential Scanning Calorimetry (DSC) curve.

#### 4.3.3. Cryo-Scanning Electron Microscopy and Energy-Dispersive X-Ray Spectroscopy

Cryo-SEM was employed for the visualization and analysis of the internal morphology and pore structure of the SUPA hydrogel network. Freshly prepared hydrogel samples were first rapidly cryo-fixed by immersion in liquid nitrogen to maximally preserve their native swollen state. Subsequently, the frozen samples were fractured mechanically to expose the internal cross-sectional morphology and then subjected to ice sublimation under high vacuum (freeze-drying). Finally, the exposed surfaces were sputter-coated with gold (or platinum) for SEM imaging. Simultaneously, Energy-Dispersive X-Ray Spectroscopy (EDS) analysis was performed on the prepared samples to confirm the content and spatial distribution of key elements, particularly silicon, thereby verifying the successful incorporation of the inorganic hybrid component (Si–O–Si nodes).

#### 4.3.4. Particle Size Analysis

The temperature-dependent particle size distribution was measured using a Mastersizer 3000 Laser Diffraction Particle Size Analyzer (Malvern Panalytical, Malvern, UK). A dilute dispersion of SUPA was circulated through a temperature-controlled cell. After a 10-min equilibration period at each step, the median particle diameter (D_50_) was recorded at discrete temperature points: 25 °C, 50 °C, 70 °C, 80 °C, and 95 °C, to quantify thermal expansion kinetics.

### 4.4. Fluid Preparation

#### 4.4.1. Preparation of 4% Na-Bentonite (Na-BT) Slurry

The 4% sodium bentonite (Na-BT) base slurry was prepared by slowly adding 16 g of sodium bentonite and 0.8 g of sodium carbonate to 400 mL of deionized water. This mixture was subjected to high-speed mechanical stirring for 20 min and subsequently aged in a sealed container at 25 °C for 24 h to ensure complete hydration. To simulate saline drilling environments, a saline base slurry was prepared by dissolving 20 g of Potassium Chloride (KCl) into the pre-hydrated 4% Na-BT slurry, yielding a final concentration of 5 wt% KCl.

#### 4.4.2. Preparation of Drilling Fluid Containing SUPA

A water-based drilling fluid was obtained from a field site in Sichuan, China. Its formulation comprised a polymer-bentonite base, barite as a weighting agent, and shale inhibitors, specifically containing 8 wt% Potassium Chloride (KCl). The fluid density was measured at 1.8 g/cm^3^. For experimental testing, SUPA-enhanced drilling fluids were prepared by adding SUPA powder to 400 mL aliquots of the field fluid at concentrations of 1.0 wt% (4 g) and 1.5 wt% (6 g), followed by high-speed stirring for 20 min to achieve uniform dispersion.

### 4.5. Rheological Measurements

#### 4.5.1. Shear Thinning Test

Rheological testing was conducted using a Haake MARS III controlled-stress rheometer (Thermo Fisher Scientific, Karlsruhe, Germany) equipped with a parallel plate geometry, with the gap distance precisely set to 0.047 mm. The steady-state shear rheological behavior (i.e., shear thinning) of the pristine field drilling fluid and the modified fluid containing 1.0 wt% SUPA was investigated. The viscosity curve was determined by logarithmically sweeping the shear rate from 1 to 1000 s^−1^. The resulting viscosity-shear rate curves were analyzed to characterize the non-Newtonian flow properties and apparent viscosity of the fluids under shear stress.

#### 4.5.2. Three-Interval Thixotropy Test

The transient three-step shear test (3ITT) was employed to assess the time-dependent rheological characteristics (thixotropy) and the kinetics of internal structure breakdown and recovery. This testing protocol comprised three consecutive stages. In the first stage (Structure Breakdown), a high constant shear rate of 1000 s^−1^ was applied for 3 min to completely disrupt the fluid’s initial microstructure. In the second stage (Structure Recovery), the shear rate was immediately reduced to 1 s^−1^ and maintained for 3 min to monitor the time-dependent recovery process of the network. Finally, in the third stage, the shear rate was increased again to 1000 s^−1^ for 3 min to quantify the degree of structural recovery and evaluate the fluid’s resistance to subsequent high shear. The change in shear stress was continuously recorded throughout the entire test.

#### 4.5.3. Oscillatory Temperature Sweep

To specifically investigate the Upper Critical Solution Temperature (UCST) phase transition behavior of the SUPA system, an oscillatory temperature sweep test was performed. Measurements were conducted at a constant frequency of 1 Hz and a non-destructive shear stress of 1 Pa (confirmed to be within the Linear Viscoelastic Region, LVR). The test procedure involved heating the sample from 25 °C to 100 °C at a constant rate of 2 °C/min, followed immediately by cooling to 25 °C at the same rate. The change in the storage modulus (G′) and loss modulus (G″) as a function of temperature was continuously monitored throughout the heating and cooling cycle.

### 4.6. Variable-Temperature UV-Vis Spectroscopy

The Upper Critical Solution Temperature (UCST) phase transition of SUPA microparticles and corresponding molecular conformational changes were precisely characterized using a UV-Vis spectrophotometer (model: UV-2600, Shimadzu, Kyoto, Japan) equipped with a Peltier temperature-controlled sample holder. The monitoring wavelength was set to 252 nm to observe the n → π∗ electronic transition of the amide C=O group within the polymer backbone. Two ultra-dilute aqueous dispersions were prepared at concentrations of 0.05 mg/mL and 0.02 mg/mL. Samples were briefly sonicated for 5 min prior to testing to ensure uniform particle dispersion. Absorbance measurements were conducted as the temperature was gradually increased from 25 °C to 95 °C. At each pre-set temperature point (25 °C, 50 °C, 70 °C, 80 °C, and 95 °C), the sample was held for 10 min to ensure thermal equilibrium and a stable polymer conformation was reached. Deionized water was used as the reference blank. Sample concentrations were strictly controlled to maintain absorbance values within the linear range of 0.2–1.0, thereby minimizing light scattering effects and effectively detecting genuine molecular absorption changes.

### 4.7. Lost Circulation Performance Testing

#### 4.7.1. Dynamic Sand Bed Filtration

Sealing capability in porous media was tested using a Model FA-1 high-temperature, high-pressure (HPHT) lost circulation testing apparatus (Qingdao Tongchun Oil Equipment Co., Ltd., Qingdao, China). 100 g of quartz sand (fine, medium, or coarse gravel) was placed in the cell. The cell was heated to the target temperature (120 °C or 150 °C). Pressure was increased by 1 MPa every 2 min up to 5 MPa. Cumulative fluid loss was recorded over 30 min at 5 MPa.

#### 4.7.2. Fracture Sealing Test (Slotted Plate)

To evaluate the sealing efficacy in fractured formations, a dynamic filtration test was conducted using a steel plate containing a precise 1 mm × 5 mm slot instead of a sand bed. The test fluid consisted of the field drilling fluid supplemented with 3 wt% mineral fiber (average length ~200 μm) and 1 wt% SUPA. The testing protocol was specifically designed to simulate field “shut-in” operations. Initially, the testing cell was heated to 150 °C. A static pressure of 1 MPa was then applied and maintained for 1 h to simulate the shut-in period. Following this, the pressure was incrementally increased by 1 MPa every 2 min until a final pressure of 5 MPa was reached. The cumulative fluid loss was recorded over a duration of 22 min at 5 MPa to assess the synergistic plugging efficiency of the SUPA-fiber system.

## Data Availability

Data is contained within the article.
